# The interaction of atoms and molecules with nanocapsules and hollow nanowires

**DOI:** 10.1038/s41598-020-72327-6

**Published:** 2020-09-24

**Authors:** Valentina A. Poteryaeva, Michael A. Bubenchikov, Alexey Michailovich Bubenchikov, Alexandr Viktorovich Lun-Fu

**Affiliations:** 1 Department of Mechanics and Mathematics, National Research Tomsk State University, Tomsk, Russia 634050; 2 Gazprom Transgaz Tomsk Ltd., Tomsk, Russia 634029; 3 Regional Scientific and Educational Mathematical Center, National Research Tomsk State University, Tomsk, Russia 634050

**Keywords:** Nanoscience and technology, Applied mathematics

## Abstract

Nanoporous membranes are widely used in various fields, such as gas separation, water purification, catalytic processes, and the use of batteries in electrodes. Nowadays, hollow carbon spheres or nanowires are attracting attention of researchers and experimenters due to high adjustability of their mechanical and chemical properties. This makes it possible, among other things, to more accurately adjust permeability of membranes created from this material for various atoms and molecules, which ensures a good degree of gas separation. The mathematical simulation of gas separation via nanocapsule and hollow nanowire porous membrane is performed. Research has shown that such membranes are able to separate He/$\text{CH}_4$/$\text{H}_2$/$\text{N}_2$ gas mixtures.

## Introduction

**Figure 1:**
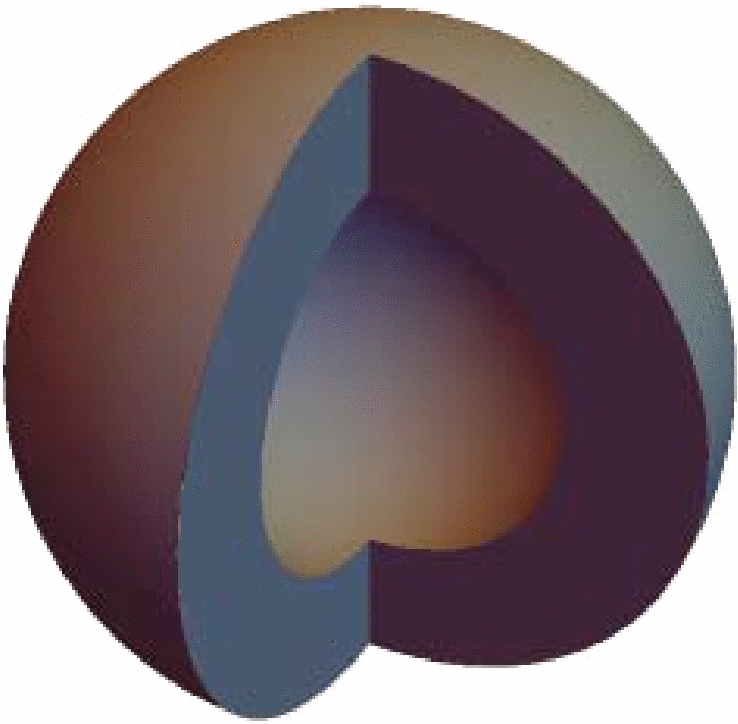
Nanocapsule.

Hollow carbon spheres, or nanocapsules, are particles of a diameter from a dozen nanometers to a millimeter^1^ with a cavity inside and a thin shell (Fig. 1). This construction provides the material with attractive encapsulation capabilities, controlled permeability, surface functionality and chemical and thermal stability. For instance, a porous shell can function as a molecular sieve providing additional selectivity and the cavity inside can be used for gas transportation^2^. Nanocapsules are incorporated into mix-matrix membranes to improve their permeability^3,4^.

The membrane method is the most attractive due to its simplicity in gas separation studies. The permeability of various carbon membranes has already been determined, but nanocapsule membranes are not often the mathematical object of research although they are promising materials. It has been shown that a nanocapsule membrane made of siliconite^5^ is able to separate the mixture $\text{H}_{2}/\text{CH}_4$. Zhang proposed a mix-matrix polymer membrane with the inclusion of hollow spheres for $\text{CO}_2/\text{N}_2$ separation^6^. In addition, membranes made of aluminum oxide^7^ and recycled soft drinks^8^ were studied to separate this mixture.

In addition to the separation of gases and liquids nanocapsules are used in biomedicine for drugs delivery^9,10^ which has an important role in the treatment of cancer^11^ as well as for the protection of proteins and enzymes^12^, as sensors^13,14^, as anode material in lithium-ion batteries^15^ and for self-healing materials^16^.

Two main techniques of synthesizing nanocapsules are distinguished according to the type of template used to create the cavity: the hard-template and the soft-template techniques. In the case of using a hard template, specially prepared solid particles are used to create a cavity, which are subsequently removed by calcination, dissolution or etching, depending on their chemical nature. The synthesis procedure usually proceeds as follows: the template is covered with a carbon precursor and then gets pyrolyzed and removed. Silicon, polymer or metal spheres can act as a template. The hard-template method allows efficiently adjusting the size of particles and their cavities; however, the process of removing the template is often complicated and not environmentally friendly.

Soft templates can be obtained by application of colloidal systems, such as emulsion droplets, micelles, vesicles or gas bubbles formed from precursor molecules or special substances originally added to form cavities. Using the soft-template technique makes it easier to remove the template; however, it provides the desired morphology and monodispersity to a lesser extent due to polydispersity and dynamics of the template^2^.

Nowadays, results of numerous successful experiments on synthesis of nanocapsules of various configurations are available. For example, the use of polymers^17^ or synthetic opal as templates^18^ allows creating macroporous capsules; work with silicon dioxide provides the material with macro-, meso- and nanopores of various morphologies^19–21^; microporous materials are obtained from zeolites and energy ashes^22–24^. In addition, nanocapsules can be produced on the basis of materials that have proven themselves as nanoporous membranes, such as porous graphenes^25^ and fullerite^26,27^.

Carbon nanowires the small sizes of which determine their unusual mechanical and electronic properties are also quite interesting structures. For the purpose of synthesising a nanowire of a specific morphology and structure it is necessary to regulate its crystallographic structure. The growth of carbon nanowires occurs according to the mechanism of carbide cycle on highly dispersed particles of metals belonging to the iron subgroup in two main stages: chemical and physical. The first stage is decomposition of hydrocarbons through formation of intermediate carbide-like compounds. Formation of crystallization centers of the graphite phase is the second stage; at this stage carbon atoms diffuse to the crystallization center and nanowires are formed. This stage determines the morphology of a nanowire.

Carbon nanowires are also an interesting structure. The small sizes of nanowires determine their unusual mechanical and electronic properties. For the synthesis of nanowires of a specific morphology and structure it is necessary to regulate its crystallographic structure. The growth of carbon nanowires occurs according to the carbide cycle mechanism on highly dispersed metal particles of the iron subgroup in two main stages: chemical and physical. The first stage is the decomposition of hydrocarbons through the formation of intermediate carbide-like compounds. At the second stage, crystallization centers of the graphite phase are formed, carbon atoms diffuse to the crystallization center, and nanowires form. This stage determines the morphology of the finished nanowire.

Carbon nanowires can have three different structural types, in which the basal graphite planes are located along (coaxial-cylindrical), across (stacked) and at a $45^{\circ }$ angle to the wire axis (coaxial-conical)^28^.

Many problems of molecular dynamics can be solved within the framework of the approach of atom-atomic interactions. This approach is that the atoms of the structure in a chemically bound state are considered as a set of free atoms that have an equivalent effect on a test atom or a moving molecule. If the question of equivalence of these effects is left aside, this approach allows the building of universal programs of molecular-dynamic calculations and, after all, the use of packet computing technologies. However, the question of the efficiency of calculations remains open. Sometimes the duration of these calculations may be excessive.

There is another approach, consisting in finding the integral effect from a representative fragment of the system (structure or a large set of fragments) on the moving particle. As in quantum mechanics, this approach allows us to determine the barrier energy of a capsule or hollow nanowire and use this information in the problems of penetration of atoms into the fragments under consideration or through a layer composed of hollow nanoelements. In this case, the nanofragment can be considered as a particle with a central or axisymmetric distribution of potential energy. Consequently, the resulting calculation programs will be simple and the calculation will be economical.

The aim of the paper is to find the symmetric interaction potentials of hollow nanoparticles and molecules (atoms), as well as to perform systematic calculations on their basis and to provide recommendations on the possibility of using the nanoparticles in sorption and membrane gas separation technologies.

## Theory and model

Let us write the basic dynamics equations of moving molecules in the Hamiltonian form^29^:

1 $$\begin{aligned} \frac{d \varvec{r_i}}{dt} = \frac{\partial H}{\partial \varvec{p_i}}, \frac{d \varvec{p_i}}{dt} = - \frac{\partial H}{\partial \varvec{r_i}} (i=\overline{1,N}). \end{aligned}$$

Here *N* is the number of particles in the molecule beam, $\varvec{r_i}$ is the vector that determine the position of the moving particle, $\varvec{p_i}$ is the particle momentum, *H* is the total energy of the system.

The Hamiltonian in the right-hand parts of Eq. (1) is defined as follows:

2 $$\begin{aligned} H=\frac{1}{2m} \sum _{i=1}^N p_i^2 + \sum _{i=1}^N \sum _{j=1}^M \Phi (\rho _{ij}), \end{aligned}$$

where *m* is the mass of moving molecule(atom), *M* is the number of components of the studied nanocapsule layer, $\rho _{ij}$ is the distance between *i*-th molecule and *j*-th capsule.

The first group of terms on the right in (2) represent the kinetic energy of the system, the second group of terms is the energy of all possible potential interactions.

The calculations below use data that corresponds to carbon nanofragments. Carbon in a chemically bound state has an effective interaction energy that is markedly less than in a free state. Therefore, the depth of the potential well in the primary LJ-potential can be reduced. At the same time, to obtain the ratio of mesomodel, the nature of potential dependence in the vicinity of zero must be changed, since the source potential used must be integrated in the volume of nanocapsule or hollow nanowire.

In general, the “nanocapsule-molecule” interaction potential can be presented as a double integral:

3 $$\begin{aligned} U(\rho _{ij})=2 \pi q \int _0^\pi {\sin \theta d\theta } \int _{a_1}^{a_2}{r'^2 P_1(sqrt(r'^2 + \rho _{ij}^2-2 r' \rho _{ij} \cos \theta ))dr'}. \end{aligned}$$

Here $a_1$ is the radius of the nanocapsule cavity; $a_2$ is the radius of the nanocapsules; *q* is the distribution density of atoms per unit volume of the substance. In particular, we can construct the particle potential on the basis of $P_1(\rho )=4 \varepsilon \dfrac{\sigma }{\rho } \tanh {\left[ (\dfrac{\sigma }{\rho })^{11} - (\dfrac{\sigma }{\rho })^5 \right] }$, the modified Lennard-Jones potential^30^. The values of the interaction constants $\sigma$ and $\varepsilon$ included in the Lennard-Jones potential for the substances considered in the work are given in Table 1.

**Table 1:** Values of the interaction constants $\varepsilon$ and $\sigma$.

Interacting molecules	The relative depth of the potential well ($\varepsilon /\kappa$), K	The influence radius of the interaction potential ($\sigma$), nm
$\text{C}{-}\text{C}$	51.2	0.343
$\text{He}{-}\text{He}$	10.22	0.2551
$\text{CH}_4{-}\text{CH}_4$	148.6	0.3758
$\text{H}_2{-}\text{H}_2$	34	0.2827
$\text{N}_2{-}\text{N}_2$	71.4	0.3798

In Table 1, *k* is the Boltzmann constant.

Due to the fact that interaction occurs between dissimilar molecules (atoms), the Lorentz-Berthelot averaging rules^31,32^ are valid for the parameters $\sigma$ and $\varepsilon$:

4 $$\begin{aligned} \sigma _{12}=\dfrac{\sigma _{11}+\sigma _{22}}{2},\ \varepsilon _{12}=\sqrt{\varepsilon _{11} \varepsilon _{22}}. \end{aligned}$$

By integrating the modified potential in two spherical angles, as well as in the radius from zero to infinity, we obtain a central-symmetric distribution of the energy of the interaction between the capsule with the moving molecule. This distribution has a potential well inside the capsule. Porous walls of the capsule determine the positive value of barrier energy. Part of the moving molecules (atoms) that have a sufficiently high energy and locate outside the capsule, overcome the energy barrier and enter the potential well, that is, inside the capsule. Calculations show that the probability of molecules coming out of the capsule is significantly less than the probability of penetration. Thus, moving particles (helium atoms) have the opportunity to be accumulated inside the capsules. The constructed mesomodel of the central-symmetric barrier layer of the capsules allows to evaluate the possibility of sorption technologies of gas separation, based on the use of hollow nanoparticles or hollow nanowires, without the use of a supercomputer.

There is a widespread technique in quantum mechanics in which the energy of a moving particle compares with the energy of a barrier. Conclusions about the passage of the particle through the barrier are made based on the result of this comparison. The same technique is used in the present work. The carbon atoms that make up the spherical layer are replaced by the energy of interaction with the moving particles representing the gas components, averaged over the thickness of this layer. Thus the average porosity, defined as the ratio of the total pores’ volume $V_{pore}$ to the volume of the entire membrane *V*:

5 $$\begin{aligned} Porosity=\frac{V_{pore}}{V}, \end{aligned}$$

is unequivocally related to the value of the average density of atoms in the structure of the capsule material.

The use of an average value does not allow taking into account the shape of the pores and the nature of their arrangement over the sphere layer of the capsule. Of course, the local density of atoms in the material of the capsule *q*(*x*, *y*, *z*) can be introduced into consideration and the mathematical model presented here is generalized to this case. However, then the barrier theory will lose its effectiveness and will not differ much from the global atomistic modeling in terms of computational costs.

The potential energy of interaction between the nanowire and the moving molecule can be calculated as follows:

6 $$\begin{aligned} U(\rho _{ij}, x_j)=2 \pi q \int _{a_1}^{a_2}{a da} \int _{-h}^{h}{P_1(\sqrt{(\rho _{ij}-a)^2 + (x_j-c)^2})dc}, \end{aligned}$$

where 2*h* is the length of the nanowire; $a_1$ is the inner radius of the nanowire; $a_2$ is the outer radius of the nanowire; $\rho _{ij}$ is the distance from the *i*-th molecule to the axis of the *j*-th nanowire; $x_j$ is the coordinate measured along the axis of the *j*-th nanowire.

Typical flat problems for the considered examples of structural elements are those corresponding to symmetric and unidirectional arrangements of these elements. Let us suppose that, in the case of a nanocapsule layer, the centers of mass of all capsules are in the same plane. In such a case, the problem of free molecular passage is two-dimensional, which is very convenient for collecting data on the statistics of molecular passage.

We will arrange nanowires parallel to each other and take such a length of each wire that in the *x* = 0 plane the influence of the distal sections of the wires is negligible. This is usually obtained at *h* = 5–7 nm.

Due to the uniform thickness and sufficient length of nanowires, when considering movements of molecules perpendicular to nanowires, only the dependence of the potential energy of a single nanowire on the distance between the center of the wire and the molecule is preserved:

7 $$\begin{aligned} U(\rho _{ij})=2 \pi q \int _{R_1}^{R_2}{a} \int _{-h}^{h}{P_1 \left( \sqrt{(\rho _{ij}-a)^2 + c^2}\right) dcda}, \end{aligned}$$

A nanocapsule (nanowire) layer is considered to be a combination of hollow spherical nanoparticles (hollow cylindrical nanowires). The potential energy of interaction between a moving particle and such a layer *U*(*z*, *y*) can be found by summing up the effects from each element of the carbon structure on the moving atom or molecule.

### Initial conditions and solution method

Equation (1) are integrated with the following initial conditions:

8 $$\begin{aligned} \varvec{r_i}|_{t=0}=\varvec{r_i}^0, \varvec{p_i}|_{t=0}=\varvec{p_i}^0 (i=\overline{1,N}). \end{aligned}$$

The index zero above indicates constant vectors.

For a numerical solution of the system of equations (1) with the initial conditions (8) the predictor-corrector method of the fourth order accuracy is used. In fact, it is the implementation of the Adams-Moulton three-step implicit method^33^. For the scalar equation of form $\frac{du}{dt} = f(u,t)$ this method can be written as follows:

9 $$\begin{aligned} \begin{aligned} {\widetilde{u}}^{k+1}&=u^k+\frac{h}{24}(55f^k-59f^{k-1}+37f^{k-2}-9f^{k-3}),\\ u^{k+1}&=u^k+\frac{h}{24}(9f^{k+1}({\widetilde{u}}^{k+1})+19f^{k}-5f^{k-1}+f^{k-2}). \end{aligned} \end{aligned}$$

Here the wave is marked the value found at the predictor step. The index above shows the values in the corresponding time layer. The method (9) has a fourth order accuracy.

All multi-step methods have an initial value problem. The method (9) is not devoid of this shortcoming. However, in the tasks of bombing the nanoparticles layer with molecules, it is solved quite simply. At the initial moment of time, moving particles (molecules or atoms) must be at such a distance from the membrane so that they can be accepted by their velocities constant, and the changes in coordinates linearly changing along rectilinear trajectories.

## Results

### Movements of atoms and molecules through a nanocapsule layer

A nanocapsule layer of particles has pores of the size of one or several nanometers, so we will randomly fill the volume leaving small gaps between them and examine the ability of test molecules to penetrate inside. The model under consideration is valid for an arbitrary sufficiently large number of particles *N*. Moreover, particles can be polydisperse, and they may be randomly distributed in space as in real situations.

**Figure 2:**
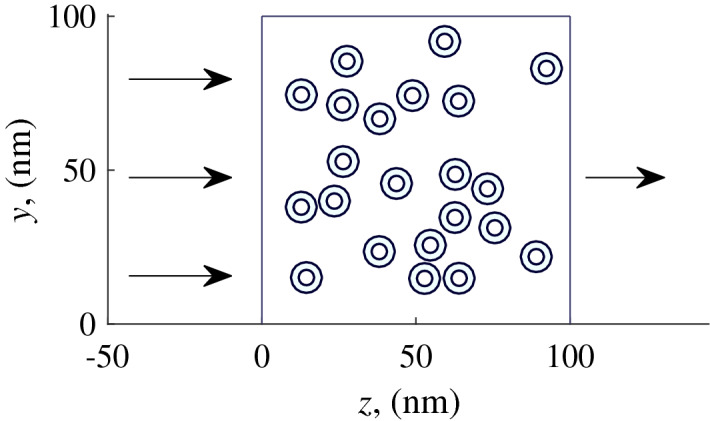
Permeable layer composed of nanocapsules or nanowires.

Let us consider a layer with a thickness of 100 nm composed of hollow nanoparticles whose center lie in the same plane 0*yz*. The origin of coordinates is placed in the lower left corner of the layer. Movements of atoms and molecules occur in the positive direction with respect to the 0*z* axis in the plane of the centers of carbon nanoparticles (Fig. 2) with the initial conditions:

10 $$\begin{aligned} \begin{aligned} \varvec{r_i}|_{t=0}&=(x_0, y_0, -10),\\ \varvec{p_i}|_{t=0}&=(0, 0, v_{T}) (i=\overline{1,N}), \end{aligned} \end{aligned}$$

where $x_0$ and $y_0$ are chosen to cover the entire region of the nanocapsule layer evenly, $v_{T}$ is the average heat velocity of the molecule at room temperature.

**Figure 3:**
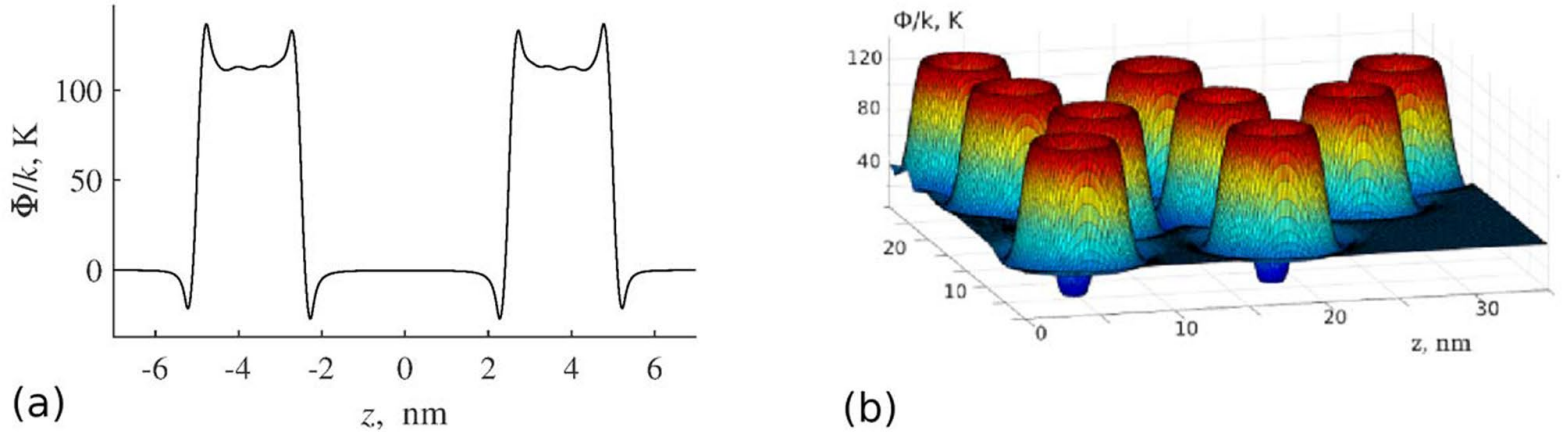
Potential energy of a hollow carbon nanocapsule of radius $a_2 = 5 \, \hbox {nm}$ and cavity radius $a_1 = 2.4 \, \hbox {nm}$ interacting with helium atom (**a**) and potential energy of a layer compacted by hollow carbon nanocapsules of radius $a_2 = 5 \, \hbox {nm}$ (**b**).

The potential energy of interaction between a single carbon nanocapsule of a radius $a_2 = 5 \, \hbox {nm}$ with a cavity radius $a_1 = 2.4 \, \hbox {nm}$ and a helium atom calculated by formula (3) is shown in Fig. 3a. Naturally, in the absence of sorption molecules, the energy barrier of the nanocapsular layer is a simple sum of potential energies of the impact of individual capsules (Fig. 3b).

**Figure 4:**
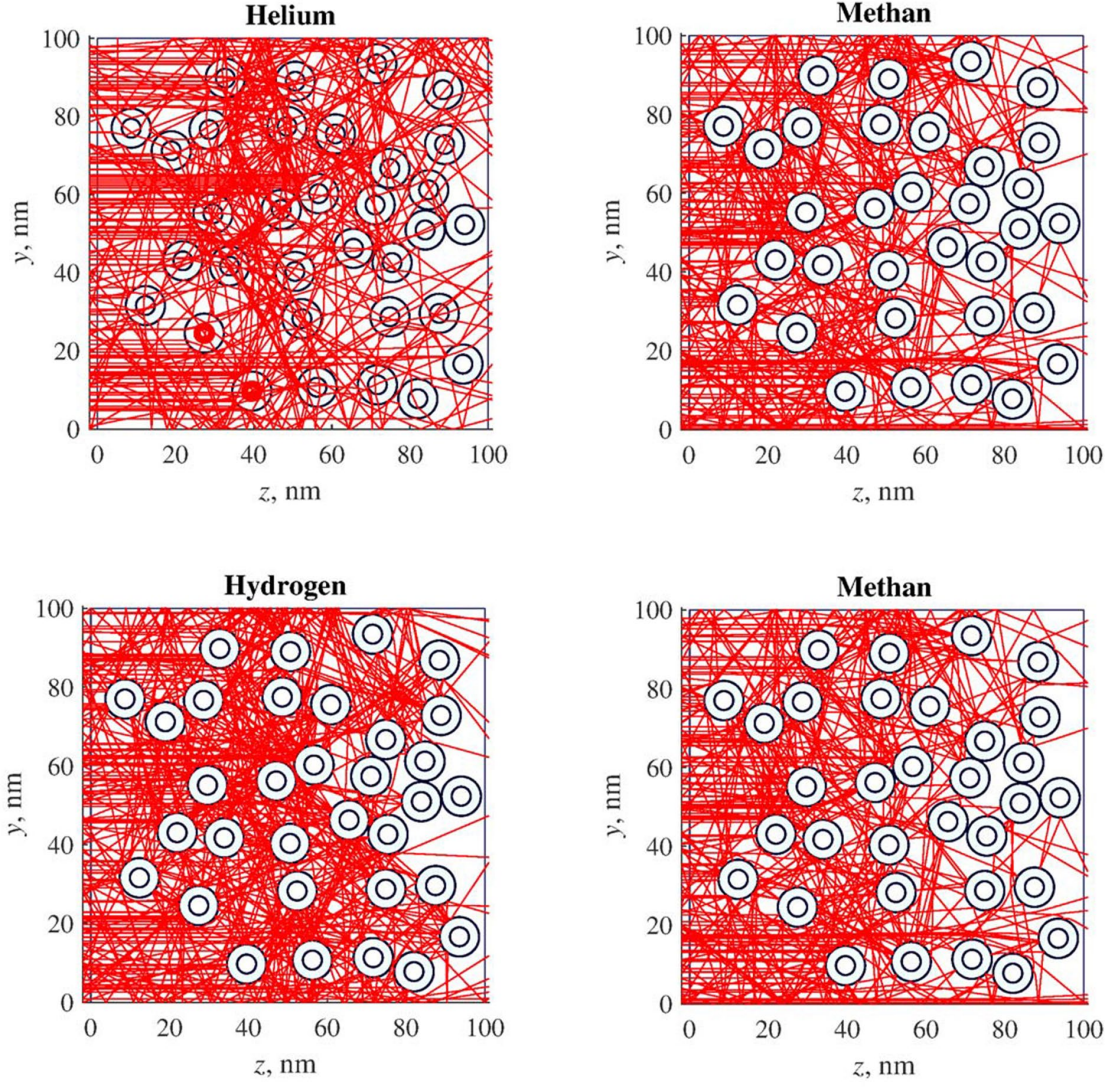
Movements of atoms and molecules through 100 nm thick nanocapsule layer.

Passage of various atoms and molecules through the considered layer is shown in Fig. 4. During the experiment we considered 100 test atoms or molecules moving from left to right. As can be seen from Fig. 4, helium atoms pass through the layer considerably better than other substances even with a sorption effect. The coefficients of the passage of helium, methane, hydrogen and nitrogen through the layer are 0.35, 0.17, 0.21, 0.15, respectively.

**Figure 5:**
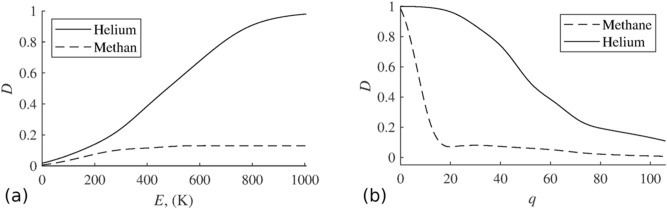
Dependence of nanocapsule layer permeability *D* on energy *E* of moving helium atom and methane molecule (**a**) and dependence of nanocapsule layer permeability *D* on its density *q* (**b**).

The dependence of the nanocapsule layer permeability *D* on the energy *E* for a moving helium atom and a methane molecule is shown in Fig. 5a. The degree of separation of gases increases with energy increasing, while methane passage set at 0.1 starting from *E*=300 K.

Within the framework of the existing structureless model, the presence of pores in the nanocapsules is taken into account only through the porosity of the shell of an individual capsule, which is characterized by the density of carbon atoms *q* in this layer. The dependence of permeability *D* of the nanocapsular layer on its density *q* is shown in Fig. 5b. As can be seen in Fig. 5b, selecting *q* from 20 to 40 provides the maximum degree of separation of the methane-helium mixture, since methane passes through such a membrane reluctantly, and there are almost no obstacles to helium passage. This value *q* corresponds to a material with a density of $6 \, {\hbox {mg/cm}}^3$. It can be a material made of ultrathin carbon fibers or nanofoam^34^.

**Figure 6:**
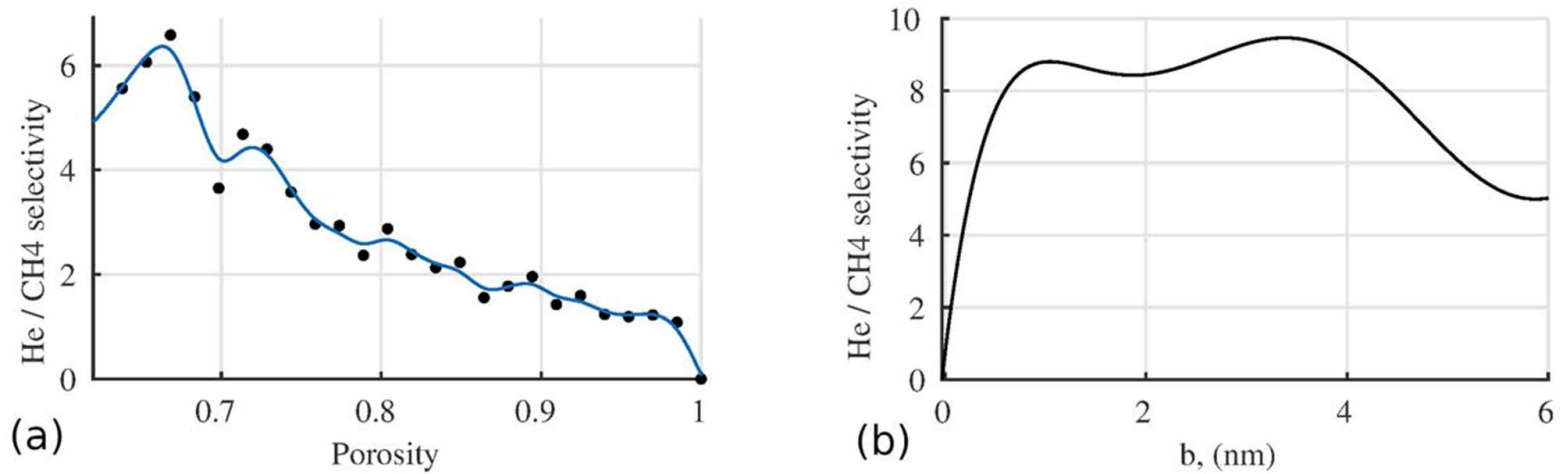
Dependence of $\text{He}/\text{CH}_4$ selectivity on *Porosity* of the layer (**a**) and on capsule shell thickness *b* (**b**).

The porosity of the membrane itself or the degree of rarefaction of nanocapsules inside the membrane layer is taken into account in Fig. 6a. As can be seen from the graph, the degree of separation decreases with increasing porosity.

The influence of the capsule wall thickness $b=R_2-R_1$ on the selectivity of separation of the $\text{He}/\text{CH}_4$ mixture passing through the capsule membrane is estimated. The outer radius of the capsule $R_2 = 6 \, \hbox {nm}$ was fixed while the inner one changed. The calculation data are shown in Fig. 6b. As can be seen, a maximum is observed in the separation intensity distribution at $b = 3.3 \, \hbox {nm}$ or $R_1 = 2.7 \, \hbox {nm}$.

### Penetration of helium atoms into a nanocapsule

At present helium is extracted from natural gas by the cryogenic method, which requires enormous expenditures of energy and material resources. One of the most promising ideas for helium separation without using low temperatures is related to the ability of light gas molecules and atoms to pass through porous structure of particular materials better than other molecules. In the case of tunnel pores, such passage can be realized in kinetic mode. Somehow, the helium atoms inside the capsule even improve the efficiency of the capsule membrane.

In the calculations presented above, the helium atoms captured by the nanocapsule were not taken into account, so the real values of the membrane permeability coefficient will be slightly higher than those shown in Fig. 5.

The ability of nanocapsule to retain helium, as well as other substances, such as drugs^9,10^, can be used for transportation and storage. Moreover, nano- and micro-capsules are embedded in the material matrix as an anticorrosion coating^35^, providing a recovery process^16^ for materials that can partially or completely repair damage, such as cracks.

**Figure 7:**
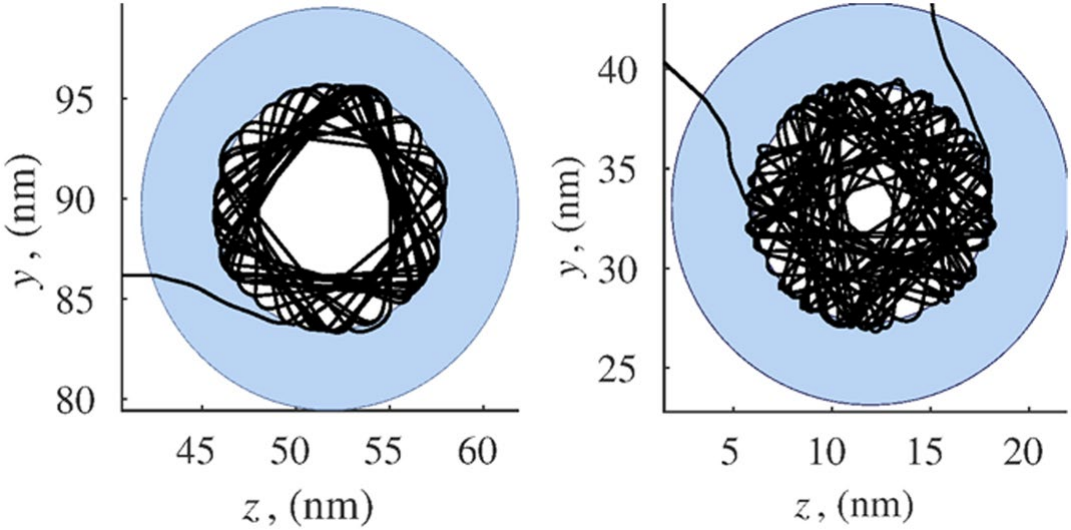
Movement of helium atoms inside a hollow spherical particle of radius $a_2 = 12 \, \hbox {nm}$ and cavity radius $a_1 = 6 \, \hbox {nm}$.

The simulation results confirm the possibility of helium atoms penetration into the hollow nanoparticle. As can be seen from Fig. 7, helium can remain inside the sphere for a long time with no suitable exit conditions. These conditions change due to systematic changes in the angle of reflection from the inner surface of the capsule, which is concave in relation to the atom inside. External atoms fall on a convex surface. Because of this, the probability of the penetration of atoms into the capsule is noticeably higher than the probability of their exit. Thus, nanocapsules of particular sizes are quite suitable for sorption technologies of helium separation.

### Movements of atoms and molecules through a nanowire layer

Let us consider a 50 nm thick layer composed of hollow nanowires. The origin of coordinates is placed in the lower left corner of the layer. The movements of atoms and molecules occur in the positive direction of the 0*z* axis perpendicular to the direction of nanowires (Fig. 2) with the initial conditions (10).

**Figure 8:**
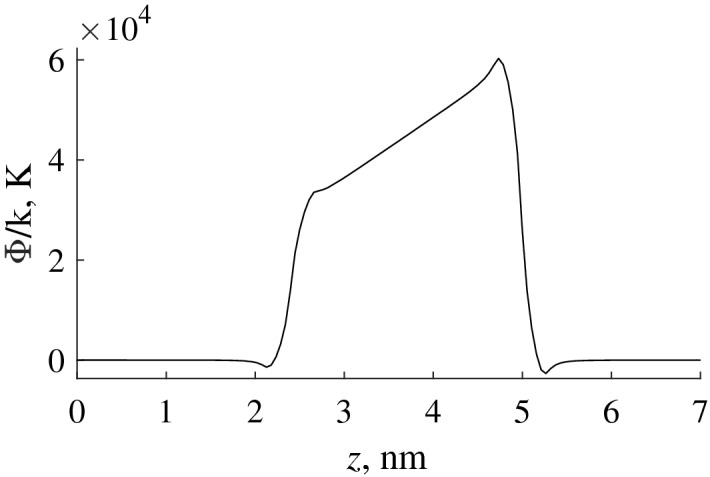
Potential energy of interaction between single carbon nanowire of radius $a_2 = 5 \, \hbox {nm}$ with internal radius $a_1 = 2$ and helium atom.

The potential energy of interaction between a single carbon nanowire of a radius $a_2 = 5 \, \hbox {nm}$ with an internal radius $a_1 = 2$ and a helium atom calculated by formula (7) is shown in Fig. 8.

After modelling of the nanowire layer is performed, the coordinates of the nanowire centers are known.

**Figure 9:**
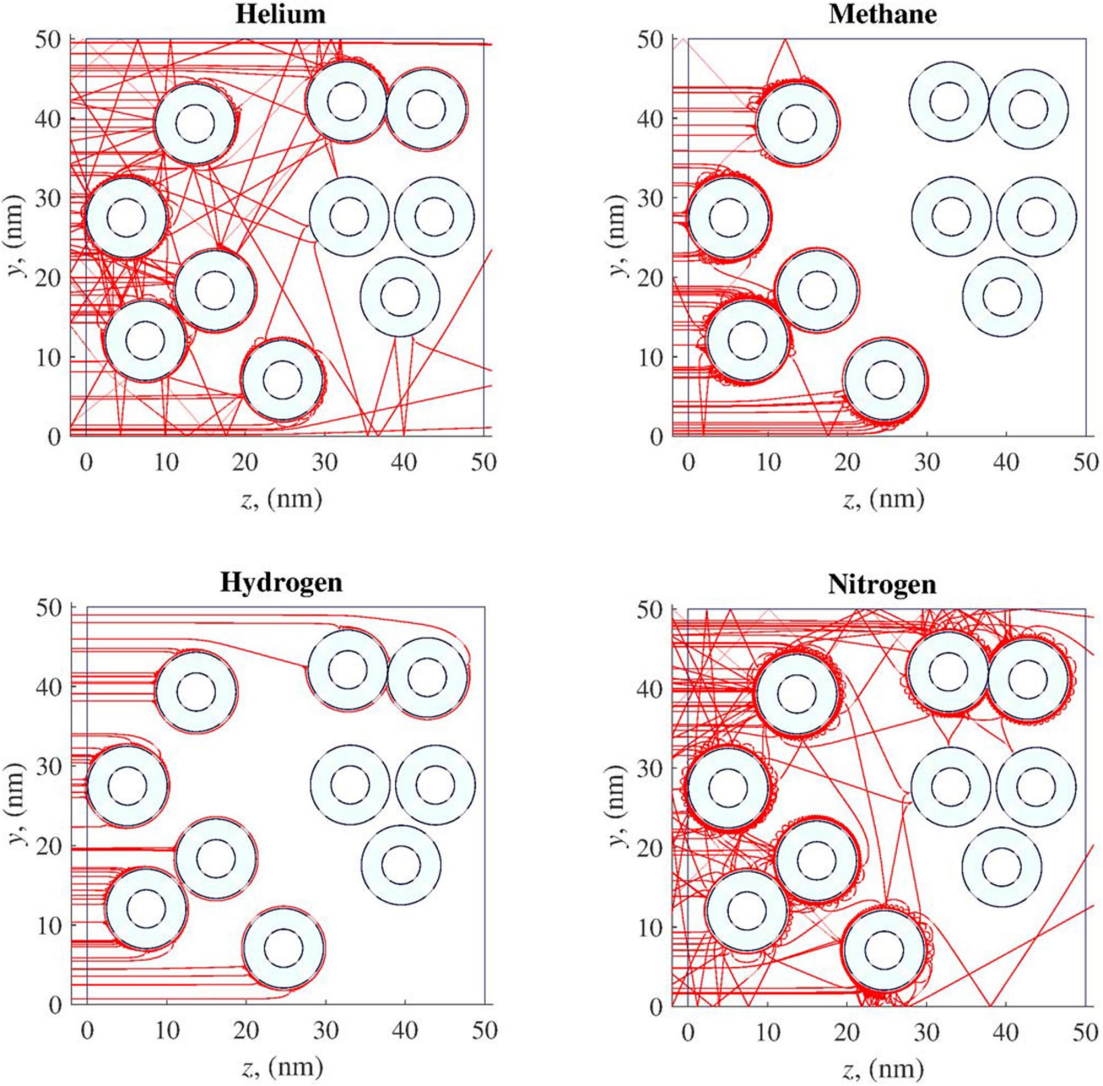
Movements of atoms and molecules through 50 nm thick nanowire layer.

Passage of various atoms and molecules through the considered layer is shown in Fig. 9. During the numerical experiment we considered 100 test atoms or molecules. As can be seen from Fig. 9, helium atoms pass through the layer better than other substances, nitrogen molecules also have a capability to pass through the layer. At the same time, methane is quickly captured by nanowires, due to the high potential energy of the interaction between $\text{CH}_4$ and a nanowire, and gets adsorbed at the surface of the wire. Hydrogen molecules are also well adsorbed by carbon nanowires. It is necessary to note another feature of the nanowires. Even helium atoms do not penetrate into them. Additionally, they are characterized by the sorption on the outer surface of the particle in relation to all the gas mixture components considered. For example, methane and hydrogen molecules enter into the sorption interaction with the nearest nanowire. According to the presented results, the separation of methane-helium mixtures with the use of nanowire carbon membranes is possible, but nanowires should have a diameter of about 10 nm, and they do not have to be hollow.

Layers of high-energy nanowires are hardly passed by gas components and are practically impermeable to methane; therefore, instead of permeability, we calculated the fraction of atoms and molecules captured by the layer (*L*) as the ratio of the number of molecules captured by the layer ($C_{captured}$) to the total number of molecules launched in its direction ($C_{launched}$) (11):

11 $$\begin{aligned} L=\frac{C_{captured}}{C_{launched}}. \end{aligned}$$

**Figure 10:**
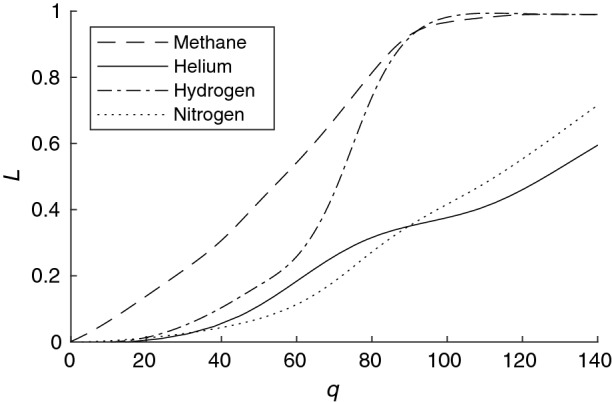
Dependence of fraction of molecules captured by nanowire layer (*L*) on density of nanowire substance *q*.

The results of these calculations depending on the density of the nanowire substance *q* are shown in Fig. 10. This graph makes it possible to conclude that the choice of a material with *q* = 90–110 can provide a good degree of helium separation from a gas mixture. For separation of methane-helium mixtures a more porous material with *q* = 70–100 would be suitable.

The foil of carbon nanowires separates nitrogen and helium from a four-component methane-hydrogen-nitrogen-helium mixture well at $q=90{-}140$.

### The trajectories and velocities of helium atoms

**Figure 11:**
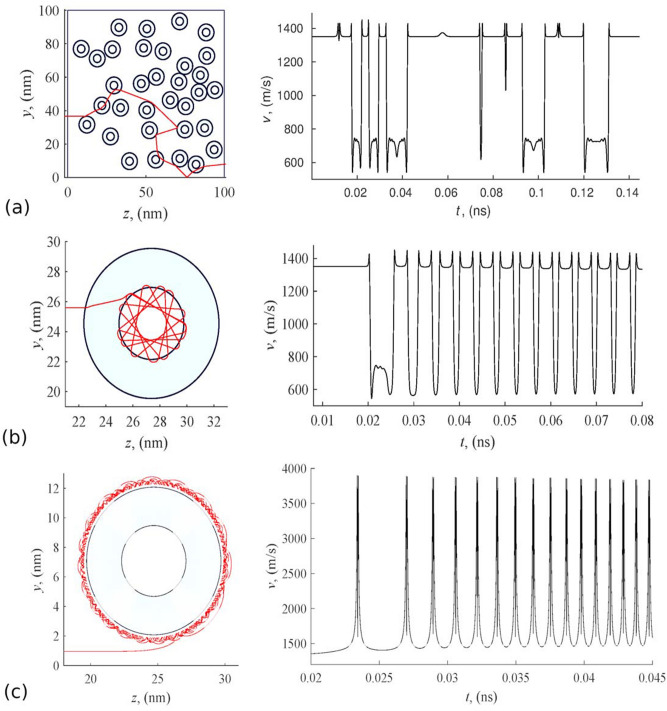
Trajectory and velocity of helium atom moving in nanocapsule layer (**a**,**b**) and trajectory and velocity of helium atom adsorbed by hollow carbon nanowire (**c**).

Figure 11 present the calculation results of molecular ballistics for helium molecules interacting with a nanocapsule layer and nanowire layers. All the data provided correspond to the mean thermal velocity of the particle at a distance from the layer. To a certain extent, this velocity represents the average characteristics of a large set of atoms distributed according to Maxwell’s law.

The left parts of the graphs in Fig. 11 show the trajectories of the helium atom. The graphs on the right represent the absolute value of velocity *v* acquired by the atom in collisions with nanocapsules. At remote distances from the system, or in zones of high local porosity, the velocity of the molecules becomes equal to the initial mean square value of thermal motion $v_T$ as in these zones the force effect from the structure becomes insignificant. Since the movements of molecules are carried out in a potential field of forces, the energy conservation law is satisfied:

12 $$\begin{aligned} \frac{mv_i^2}{2}=\sum _{j=1}^n U(\rho _{ij})+\frac{mv_T^2}{2}, i=\overline{1,N}. \end{aligned}$$

Relations (12) are the first integral of the equations of motion given in (1) and can be used to control the accuracy of calculations.

All the calculations presented were carried out at room temperature, that is, 300 K. Within certain limits, the temperature can be increased or decreased by correspondingly changing the average velocity of the particle beam bombarding the capsule membrane. In this case, however, all calculations must be performed anew.

The calculations presented refer to membranes formed by identical hollow nanowires and capsules. However, the mathematical model is quite economical, and polydisperse membranes can also be analyzed for permeability and selectivity in relation to any mixtures.

## Conclusions

Membranes composed of hollow nanoparticles can be successfully used to separate atoms and molecules. Unlike those which are uniformly filled with carbon molecules, hollow particles present a possibility to more accurately adjust permeability of membranes.

Based on the conducted calculations we can conclude that at room temperature nanocapsule membranes composed of particles with a density of matter *q* = 20–40 are an effective means of separation for methane-helium mixtures.

Moreover, carbon capsules that pass helium into the inner cavity and do not pass molecular components, including light hydrogen ($\text{H}_2$), can be used as sorbing elements in the corresponding technologies. The internal cavities of carbon nanowires are unattainable even for helium atoms. But sorption on the outer surface of nanowires with a diameter of about 10 nm is realized for all the components considered. Therefore, nanofiber carbon materials can be a good sorbent for a number of gas mixtures components. At the same time, the foil of such wires captures methane and molecular hydrogen completely, allowing helium and nitrogen to pass through.

## References

[1] Lee J, Kim J, Hyeon T (2006). Recent progress in the synthesis of porous carbon materials. Adv. Mater..

[2] Li S, Pasc A, Fierroa V, Celzard A (2016). Hollow carbon spheres, synthesis and applications - a review. J. Mater. Chem. A.

[3] Ding X (2019). Enhancing gas permeation and separation performance of polymeric membrane by incorporating hollow polyamide nanoparticles with dense shell. J. Membr. Sci..

[4] Hwang S (2015). Hollow ZIF-8 nanoparticles improve the permeability of mixed matrix membranes for $$CO_2$$/$$CH_4$$ gas separation. J. Membr. Sci..

[5] Zornoza B, Esekhile O, Koros WJ, Téllez C, Coronas J (2011). Hollow silicalite-1 sphere-polymer mixed matrix membranes for gas separation. Sep. Purif. Technol..

[6] Zhang J (2016). Membrane-based gas separation accelerated by hollow nanosphere architectures. Adv. Mater..

[7] Simonato S (2012). Sorption and separation of $$CO_2$$ via nanoscale AlO(OH) hollow spheres. Chem. Commun..

[8] Teague CM (2019). Microporous and hollow carbon spheres derived from soft drinks: Promising $$CO_2$$ separation materials. Microporous Mesoporous Mater..

[9] Chen Y (2014). Colloidal RBC-shaped, hydrophilic, and hollow mesoporous carbon nanocapsules for highly efficient biomedical engineering. J. Adv. Mater..

[10] Chen Y (2013). Colloidal HPMO nanoparticles: Silica-etching chemistry tailoring, topological transformation, and nano-biomedical applications. J. Adv. Mater..

[11] Mezni A (2017). Highly biocompatible carbon nanocapsules derived from plastic waste for advanced cancer therapy. J. Drug Deliv. Sci. Technol..

[12] Wen J (2011). Controlled protein delivery based on enzyme-responsive nanocapsules. Adv. Mater..

[13] Marsh H (1989). Introduction to Carbon Science.

[14] Jang J, Lim B (2002). Selective fabrication of carbon nanocapsules and mesocellular foams by surface-modified colloidal silica templating. Adv. Mater..

[15] Liu X (2013). $$Co_3O_4/C$$ nanocapsules with onion-like carbon shells as anode material for lithium ion batteries. Electrochim. Acta.

[16] Blaiszik BJ, Sottos NR, White SR (2008). Nanocapsules for self-healing materials. Compos. Sci. Technol..

[17] Tamai H, Sumi T, Yasuda H (1996). Preparation and characteristics of fine hollow carbon particles. J. Colloid Interface Sci..

[18] Zakhidov AA (1998). Carbon structures with three-dimensional periodicity at optical wavelengths. Science.

[19] Johnson SA, Ollivier PJ, Mallouk TE (1999). Ordered mesoporous polymers of tunable pore size from colloidal silica templates. Science.

[20] Yu J-S, Kang S, Yoon SB, Chai G (2002). Fabrication of ordered uniform porous carbon networks and their application to a catalyst supporter. J. Am. Chem. Soc..

[21] Xia Y, Mokaya R (2004). Ordered mesoporous carbon hollow spheres nanocast using mesoporous silica via chemical vapor deposition. Adv. Mater..

[22] Chernih YY, Vereshagin SN (2011). Helium permeability studies of fly ash cenospheres. J. Sib. Fed. Univ..

[23] Kim M (2003). Synthesis and characterization of spherical carbon and polymer capsules with hollow macroporous core and mesoporous shell structures. Microporous Mesoporous Mater..

[24] Yoon SB (2002). Fabrication of carbon capsules with hollow macroporous core/mesoporous shell structures. Adv. Mater..

[25] Bubenchikov AM (2016). The effect of graphene shape on its ability to separate gases. Russ. Phys. J..

[26] Bubenchikov MA, Bubenchikov AM, Tarasov EA, Usenko OV, Chelnokova AS (2018). Calculating permeability of the low-temperature phase of a fullerite. Diam. Relat. Mater..

[27] Manna AK, Pati SK (2013). Stability and electronic structure of carbon capsules with superior gas storage properties: A theoretical study. Chem. Phys..

[28] Chesnokov VV, Buyanov RA (2005). Peculiarities of formation of carbon nanofilaments with various crystallographic structure from hydrocarbons over the catalysts containing iron subgroup metals. Crit. Techol. Membr..

[29] Landau LD, Lifshitz EM, Pitaevsky LP (1988). The canonical equations. Theoretical Physics Mechanics.

[30] Bubenchikov AM, Bubenchikov MA, Potekaev AI, Libin EE, Khudobina YP (2015). The potential field of carbon bodies as a basis for sorption properties of barrier gas systems. Russ. Phys. J..

[31] Lorentz HA (1881). Ueber die Anwendung des Satzes vom Virial in der kinetischen Theorie der Gase. Annalen der Physik.

[32] Berthelot D (1898). Sur le mélange des gaz. Comptes rendus hebdomadaires des séances de l'Académie des Sciences.

[33] Quarteroni A, Sacco R, Saleri F, Marsden JE (2007). Numerical solution of ordinary differential equations. Numerical Mathematics. Texts in Applied Mathematics.

[34] Mainak, R. Nanocrystalline and disordered carbon materials. In *Functional Materials. Preparation, Processing and Applications*, 675–706 (2012).

[35] Judit T, Abdul S, Gyöngyi V (2018). Micro/nanocapsules for anticorrosion coatings in fundamentals of nanoparticles.

